# Endosomal Phosphoinositides and Human Diseases

**DOI:** 10.1111/j.1600-0854.2008.00754.x

**Published:** 2008-05-20

**Authors:** Anne-Sophie Nicot, Jocelyn Laporte

**Affiliations:** Department of Neurobiology and Genetics, Institut de Génétique et de Biologie Moléculaire et Cellulaire, INSERM U596, CNRS UMR 7104, Université Louis Pasteur de Strasbourg, Collège de France67404 Illkirch, France

**Keywords:** centronuclear myopathy, Charcot–Marie–Tooth peripheral neuropathy, endosome, FIG4, membrane remodeling, myotubularin, myotubular myopathy, phosphoinositides, PI 3-kinase, PIK3C3, PIKfyve, PIP5K3, SAC3, VPS34

## Abstract

Phosphoinositides (PIs) are lipid second messengers implicated in signal transduction and membrane trafficking. Seven distinct PIs can be synthesized by phosphorylation of the inositol ring of phosphatidylinositol (PtdIns), and their metabolism is accurately regulated by PI kinases and phosphatases. Two of the PIs, PtdIns3*P* and PtdIns(3,5)*P*_2_, are present on intracellular endosomal compartments, and several studies suggest that they have a role in membrane remodeling and trafficking. We refer to them as ‘endosomal PIs’. An increasing number of human genetic diseases including myopathy and neuropathies are associated to mutations in enzymes regulating the turnover of these endosomal PIs. The PtdIns3*P* and PtdIns(3,5)*P*_2_ 3-phosphatase myotubularin gene is mutated in X-linked centronuclear myopathy, whereas its homologs MTMR2 and MTMR13 and the PtdIns(3,5)*P*_2_ 5-phosphatase SAC3/FIG4 are implicated in Charcot–Marie–Tooth peripheral neuropathies. Mutations in the gene encoding the PtdIns3*P*5-kinase PIP5K3/PIKfyve have been found in patients affected with François–Neetens fleck corneal dystrophy. This review presents the roles of the endosomal PIs and their regulators and proposes defects of membrane remodeling as a common pathological mechanism for the corresponding diseases.

In eukaryotic cells, spatiotemporal regulation of cellular organization and cell–cell communication require tightly regulated second messengers and membrane microdomains. Phosphoinositides (PIs) are lipid second messengers implicated in signal transduction and membrane trafficking through recruitment of protein effectors to their site of action (1). Phosphatidylinositol (PtdIns) is one of the major lipids within the cell and is composed of different chemical moieties: two fatty acid tails anchor the PtdIns molecule into cellular membranes and are linked through a glycerol backbone and an inorganic phosphate to the inositol head group. This inositol sugar ring directs into the cellular lumen and can be phosphorylated on positions three, four and five, resulting in seven possible PIs. Interconversions between different PIs are controlled by PI kinases and phosphatases. A plethora of reports showed their involvement in signal transduction, but pioneer studies, especially in yeast, have shown that among the various PIs, PtdIns3*P* and PtdIns(3,5)*P*_2_ are involved in membrane trafficking (2,3). We will focus here on these so-called ‘endosomal PIs’, PtdIns3*P* and PtdIns(3,5)*P*_2_. PtdIns3*P* was first discovered through the characterization of PI kinases in fibroblasts and by radioactive labeling of astrocytoma cells, while studies in osmotically stressed yeast strains and in resting mouse cells led to the detection of *in vivo* PtdIns(3,5)*P*_2_ pools (4–7). Their regulators are implicated in human genetic diseases including several neuromuscular disorders. The impact of PtdIns3*P* and PtdIns(3,5)*P*_2_ metabolism unbalance on the phenotypic manifestations remains poorly understood, but one may hypothesize that the diseases share some common pathomechanisms involving abnormal trafficking. Phenotypes seen in patients may thus highlight new roles of PIs and implicated regulators, and conversely, the known roles of PIs and regulators may help to understand the pathophysiology of the related diseases.

The canonical PIs turnover pathway implicates the phosphorylation of PtdIns into PtdIns3*P* by the class III PI 3-kinase (PIK3C3, also called VPS34 in *Saccharomyces cerevisiae*). PtdIns3*P* is subsequently phosphorylated by the type III PI 5-kinase (PIP5K3, also called PIKfyve, and FAB1 in *S. cerevisiae*) into PtdIns(3,5)*P*_2_, the reverse reaction being catalyzed by the phosphatase SAC3 (the ortholog of *S. cerevisiae* FIG4). PtdIns(3,5)*P*_2_ can be dephosphorylated by the 3-phosphatase myotubularins (MTMs), leading to the production of PtdIns5*P*. Myotubularins also dephosphorylate PtdIns3*P* into PtdIns (Figure 1). Other alternative pathways have been suggested by *in vitro* experiments and may exist *in vivo*, leading to the production of PtdIns3*P* and PtdIns(3,5)*P*_2_ through the action of class II PI 3-kinase, 4-phosphatase and 5-phosphatase acting on PtdIns(3,4,5)*P*_3_ or class I PI 3-kinase acting on PtdIns5*P*, respectively (8,9).

**Figure 1:**
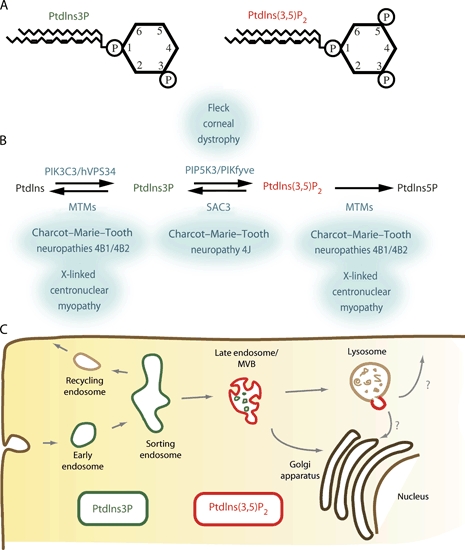
Endosomal PI structure, metabolism, subcellular localization and associated human diseases A) Chemical structure of PtdIns that can be phosphorylated on position 3, 4 and/or 5. B) Interconversion of PtdIns3*P* and PtdIns(3,5)*P*_2_ is catalyzed by kinases, PIK3C3 (VPS34 in *Saccharomyces cerevisiae*) and PIP5K3 (also called PIKfyve and FAB1 in *S. cerevisiae*), and phosphatases, SAC3 (FIG4 in *S. cerevisiae*) and myotubularins, including MTM1, MTMR2 and the inactive phosphatase MTMR13. Associated human diseases are shown in blue. C) Subcellular localization of PtdIns3*P* (in green) and PtdIns(3,5)*P*_2_ (in red) are shown in the endocytic pathway: early endosomes, sorting endosomes, recycling endosomes for receptor recycling and MVBs/late endosomes and lysosomes for degradation. The localization of PtdIns(3,5)*P*_2_ is hypothetical. Based on works performed in yeast, *Drosophila*, *Caenorhabditis elegans* and mammalian cells, PtdIns(3,5)*P*_2_ should be present at the external membrane of late endosomes and at lysosomes. The destiny of lysosomes is not known but rather hypothetical based on the same studies.

## Production of PtdIns3*P* by PIK3C3/hVPS34, a Candidate for Schizophrenia and Autophagy-Related Diseases

PtdIns3*P*, the product of PIK3C3, is involved in membrane transport and dynamics. It is highly enriched on early endosomes and in the internal vesicles of multivesicular bodies (MVBs) and can recruit proteins containing FYVE, PX or PH motifs (10). Among them, early endosome antigen 1 (EEA1), a protein essential for endosome fusion, is recruited to early endosomes by Rab5-GTP and PtdIns3*P* (11). Moreover, the complex formed by PIK3C3 and its regulatory subunit PIK3R4 (also called p150 and VPS15 in *S. cerevisiae*) produces PtdIns3*P* and binds Rab5 (12). Hepatocyte growth factor regulated tyrosine kinase substrate (HRS), another FYVE domain protein, is recruited to early endosomes/MVBs by PtdIns3*P* and controls the first steps of receptor sorting and internalization within the MVBs (13–15). VPS34 also regulates protein sorting to the lysosome in addition to vacuole segregation during mitosis in *S. cerevisiae* and is involved in membrane homeostasis as shown by the enlarged vacuoles of *Schizosaccharomyces pombe* strains deleted for the *Vps34* gene and in cultured mammalian cells treated with small interfering RNA (siRNA) against *PIK3C3* (2,16–18). In addition to PtdIns3*P* roles and localization at intracellular compartments, a regulated pool of PtdIns3*P* is specifically formed at the plasma membrane of cultured muscle cells upon insulin stimulation through activation of the small GTP-binding protein TC10 and is involved in the translocation of the glucose transporter protein GLUT4 to the plasma membrane (19). Production of PtdIns3*P* by PIK3C2A may also mediate ATP-dependent priming of neurosecretory granule exocytosis (20). Thus, PtdIns3*P* could be involved in the translocation of storage compartments under stimulation. Studies on *Caenorhabditis elegans* also point to a role of PIK3C3 and PtdIns3*P* at plasma membrane and nuclear membrane as deletion of *CePIK3C3* results both in endocytosis defect and in expansion of the outer nuclear membrane (21,22). Therefore, PtdIns3*P* and PIK3C3 are required for membrane recruitment of several proteins involved in the control of vesicular transport and intracellular protein sorting.

Moreover, it is proposed that PIK3C3 and its product PtdIns3*P* are involved in the control of autophagy vesicles. This is supported by the finding that PI 3-kinase inhibitors impair autophagy, that exogenous PtdIns3*P* rescues these autophagy defects and that PIK3C3 interacts with proteins involved in the autophagy process like Beclin 1 (23,24). Autophagy is altered in many human diseases like cancer, some myopathies where autophagic vacuoles accumulate (for example in Danon's disease) and a number of neurodegenerative disorders where the accumulation of misfolded proteins could result from a defect in autophagy (25). No causative mutation in *PIK3C3* has been found yet in these types of diseases. However, three accessory subunits of the PIK3C3–PIK3R4 complex are tumor suppressors (Beclin 1, Uvrag and Bif-1) (26). A rare variant in the promoter of *PIK3C3* gene has been reported to be associated with bipolar disorder and schizophrenia in a candidate gene study (27,28). This variant occurs within an octamer sequence containing an ATTT core motif found in promoters that bind to members of the Pit-1, Oct, unc-86 (POU) transcription factor family and was observed to induce binding of an unidentified nuclear protein by gel shift assay (28). However, the association data appear rather confusing in different populations; statistical significance was not corrected for multiple testing and the association to bipolar disorder or schizophrenia should thus be considered tentative at best. A link between PIK3C3 and bipolar disorder might be consistent with the observation that PIs are potential targets for the therapeutic effect of lithium in bipolar disorder.

## Production of PtdIns(3,5)*P*_2_ by PIP5K3/PIKfyve and Corneal Fleck Dystrophy

Heterozygous mutations of the *PIP5K3* gene have been found in cases of autosomal dominant François–Neetens corneal fleck dystrophy (CFD, OMIM 121850), which is characterized by abnormal swollen keratocytes with enlarged vesicles of unknown origin in the cornea (29). Except for an occasional patient with minor photophobia, patients have a normal vision. The majority of mutations found in François–Neetens CFD are nonsense or frameshift heterozygous mutations that result in a protein deleted for its kinase domain. Whether this leads to a dominant negative effect or a haploinsufficiency has not been determined yet (29).

An important implication of PIP5K3 in vacuole size regulation has been underlined. Indeed, the yeast vacuole, analogous to the mammalian lysosome, is enlarged in *Fab1* mutants (3,30). FAB1 is important for vacuole size regulation, protein sorting at MVB and vacuole acidification (31,32). Mutants deleted for *Vac7* or *Vac14*, which encode two known activators of FAB1, recapitulate enlarged vacuoles and acidification defects of *Fab1*-deleted strains (33,34). Overexpression of a kinase-defective PIP5K3 mutant, suppression of PIP5K3 by siRNA and drug-specific inhibition of PIP5K3 enzymatic activity produce abnormal vesicles described as enlarged early/late endosomes (35–37). PIP5K3 suppressed cells, as well as *Drosophila* and *C. elegans PIP5K3* mutants where enlarged late endosomes/lysosomes are also present, do not show receptor signaling and protein degradation defect, respectively, suggesting that PIP5K3 has a role in the retrieval of membranes exiting the endosomal pathway from early and late endosomes and lysosomes (37–39). Indeed, Rutherford et al. observed that PIP5K3 regulates endosome-to-*trans* Golgi network (TGN) retrograde transport and PIP5K3 interacts with p40, a RAB9 effector implicated in retrograde traffic from the late endosome (37,40). This mechanism could be mediated through a putative role of PtdIns(3,5)*P*_2_ in membrane fission as suggested in wild-type yeast where PtdIns(3,5)*P*_2_ levels increase upon hyperosmotic shock and lead to vacuolar fragmentation (4,33). Subcellular localization of PtdIns(3,5)*P*_2_ is still unclear because of the lack of well-characterized tools to detect it and is based on indirect evidences obtained in yeast, *Drosophila*, *C. elegans* and mammalian cells. Part of the roles of PIP5K3 are transduced by PtdIns(3,5)*P*_2_ effectors (41). These include Epsin N-terminal homology 3 (ENT3), ENT5 and vacuolar protein sorting 24 (mVPS24) for MVB sorting and autophagy related 18 (ATG18) for membrane retrograde transport and partitioning of the vacuole (42–44). ATG18 has also been implicated in membrane and protein retrieval from the preautophagosomal structures (45). PIP5K3 has been described as a regulator of insulin-stimulated GLUT4 vesicle translocation to the plasma membrane following its phosphorylation by protein kinase B and as an inhibitor of exocytosis in neurosecretory cells (46,47). Therefore, numerous organisms recapitulate the CFD phenotype of vesicle enlargement. Part of the underlying cause of this disease may be a defect in membrane fission or retrieval from endosomes and membrane storage compartments. Heterozygous *PIP5K3* mutations result in a mild phenotype in the eye. It is not excluded that other tissues contain vacuoles in CFD patients, and PIP5K3 homozygous mutations may be found in more invalidating diseases.

## PtdIns3*P* and PtdIns(3,5)*P*_2_ Dephosphorylation and Charcot–Marie–Tooth Diseases

Charcot–Marie–Tooth diseases (CMT) are a heterogeneous group of genetic peripheral neuropathies affecting motor and sensory nerves and are characterized by progressive distal muscle weakness and atrophy. Nerve conduction velocity (NCV) tests are used to differentiate between the demyelinating forms of CMT (types 1, 3 and 4; forearm motor NCV ≤ 38 m/second) and the axonal form of CMT (type 2; NCV ≥ 38 m/second). Autosomal recessive forms of demyelinating CMT are collectively designated CMT4 (48). Nerve biopsies from CMT4B patients are characterized by remarkable focally folded myelin sheaths (49). Myelin in the peripheral nervous system is generated and maintained by myelinating Schwann cells. This highly specialized cell type enwraps segments of axons with multiple layers of its plasma membrane.

### SAC3/FIG4 and CMT4J neuropathy

Compound heterozygous mutations of the human *SAC3* gene have been identified in four unrelated patients with autosomal recessive CMT neuropathy named CMT4J (OMIM 611228) (50). These patients presented one allele encoding an inactivating missense mutation I41T upstream of the SAC phosphatase domain, while the other allele carried a truncating mutation located upstream or within the SAC domain.

The identification of *SAC3* mutations has been allowed by the observation of phenotype similarities between CMT patients and the ‘pale tremor mouse’. This mouse contains an insertion of a transposon into an intron of the *SAC3* gene resulting in a loss of the protein and presents neuronal degeneration in the central nervous system, peripheral neuropathy and diluted pigmentation. Cultured fibroblasts from pale tremor mice are filled with enlarged late endosomes/lysosomes and show a reduced level of PtdIns(3,5)*P*_2_ compared with wild type in apparent discrepancy with the PtdIns(3,5)*P*_2_ 5-phosphatase function of SAC3 (50,51). This phenotype mimics PIP5K3/FAB1 suppression and is similar to *FIG4*-deficient yeast strains (52). There are several possible explanations for the unexpected PtdIns(3,5)*P*_2_ decrease following *SAC3* mutations. SAC3 interacts with one PIP5K3 activator VAC14 and has been shown to be required for the hyperosmotic shock-induced elevation of PtdIns(3,5)*P*_2_ levels (51,53,54). The absence of SAC3 may then result in defects in VAC14-dependent PIP5K3 activation, as sustained by the similar phenotype of the mouse mutant lacking VAC14 (55). Alternatively, SAC3 may be a direct activator of PIP5K3. The SAC3 I41T mutant found in CMT4J patients is unable to fully activate PIP5K3 kinase activity in a complementation assay of *FIG4*-deleted yeast strains subjected to hyperosmotic shock (50). In the pale tremor mice, peripheral nerves are affected and vacuoles accumulation precedes cell loss in the central nervous system. Functional analysis suggests that such vacuoles could originate from a lack of retrograde transport from late endosomes/lysosomes, similarly to CFD. Neurodegeneration may be related to the role of endosomal vesicles in delivering membrane components to dendritic spines or, if degradation is affected, to a deleterious accumulation of proteins. In the peripheral nervous system, in addition to axonal degeneration, it is expected that impaired conduction velocity observed in pale tremor mice and in CMT4J patients reflects defects in myelin sheath formation, as it is the case in other forms of demyelinating CMT.

### MTMR2 and CMT4B1 demyelinating neuropathy

The gene coding for myotubularin-related protein-2 (MTMR2) was found mutated in autosomal recessive demyelinating neuropathy CMT type 4B (CMT4B1, OMIM 601382) (56). MTMR2 is part of the large family of myotubularins, conserved in yeast, and encompassing 14 members in human (myotubularin MTM1 and myotubularin-related proteins MTMR1 to 13) (57). Eight of the human myotubularins are 3-phosphatases sharing specificity toward PtdIns3*P* and PtdIns(3,5)*P*_2_(58–60). The myotubularin family also encompasses inactive phosphatases that lack key residues in the catalytic loop but are conserved through evolution. CMT4B1-causing mutations in *MTMR2* are located all along the gene, giving rise to truncated or inactive proteins (56,61).

*Mtmr2* knockout mouse models develop a mild CMT4B1-like neuropathy and azoospermia. Myelination of peripheral nerves is abnormal in the knockout mice, with myelin outfoldings and recurrent loops originating at paranodal regions (62–64). Schwann cell-specific ablation of *Mtmr2* is sufficient to mimic the CMT4B1 phenotype in mice, while motoneuron-specific ablation has no effect, strongly suggesting that the myelinating Schwann cell is initially affected in CMT4B1 (62,63). Moreover, MTMR2 has recently been reported to play a role in cell proliferation and survival of Schwann cells in primary cultures (65). Overexpression of human MTMR2 in yeast leads to enlarged vacuoles and, in hypoosmotically stressed COS cells with increased levels of PtdIns(3,5)*P*_2_, MTMR2 is recruited to the membrane of vacuoles formed under these conditions (66,67). This redistribution of MTMR2 is dependent on its PH-GRAM domain, which has been proposed to bind PtdIns(3,5)*P*_2_ and/or PtdIns5*P* (68–70). These data suggest that endogenous MTMR2 could be implicated in vacuolar transport and fusion through its phosphatase activity toward PtdIns(3,5)*P*_2_ and PtdIns3*P*. MTMR2 may be regulated by lipid and protein interactors. The PH-GRAM domain of several MTMR2 homologs mediates a PtdIns3*P*/PtdIns5*P*-dependent oligomerization and a PtdIns5*P*-specific activation (71). Moreover, MTMR2 is also regulated by heterodimerization with inactive phosphatase homologs, MTMR5/SBF1 and MTMR13/SBF2 (72–74). *MTMR13* is also mutated in CMT4B2, suggesting that MTMR2/MTMR13 interaction is important in Schwann cells. Interestingly, the neurofilament light chain NF-L, mutated in dominant axonal CMT2E or dominant demyelinating CMT1F, interacts with MTMR2 in Schwann cells as well as in neurons (75,76). MTMR2 also binds to discs large 1 (Dlg1)/synapse-associated protein 97 (SAP97) in myelinated nerve fibers (62). Dlg1/SAP97, a membrane associated guanylate kinase-like (MAGUK), is a scaffolding protein specifically detected in Schwann cells at the node/paranodal region where myelin outfoldings and recurrent loops originate in CMT4B1 (62). Dlg1 homologs have been located in several types of cellular junctions and play important roles in cell polarity and membrane addition (77). Loss of MTMR2/Dlg1 interaction in Schwann cells may impair membrane homeostasis in the paranodal region, thereby leading to myelin defects. The functions of MTMR2 and of its inactive partner MTMR5 may also be important in testis as both MTMR2 and MTMR5 ablations in mice lead to defects in spermatogenesis (62,78). It has been proposed that *Mtmr2*-deleted mice show azoospermia because of a loss of cell adhesion between Sertoli and germ cells within the seminiferous epithelium (62). This is consistent with the finding that MTMR2 interacts with *c-src*, a non-receptor protein tyrosine kinase that is part of the *N*-cadherin/β-catenin functional complex at adherens junctions (79). These findings support the idea that the association between MTMR2 and MTMR5 could be of physiological significance in spermatogenesis.

### MTMR13 and CMT4B2 demyelinating neuropathy

Mutations in the *MTMR13/SBF2* gene, which encodes a catalytically inactive member of the myotubularin family involved in heterodimerization with MTMR2, cause CMT4B2 (OMIM 604563), which has similar pathological features to CMT4B1, with additional glaucoma in some patients (80,81). Five distinct CMT4B2-causing mutations have been reported in the *MTMR13* gene and are predicted to result in the loss of MTMR13 protein or to a truncated product (80–82).

*Mtmr13* knockout mice reproduce myelin outfoldings in peripheral nerves as the pathological hallmarks of CMT4B2 (83,84). The physical interaction between MTMR2 and MTMR13 offers a molecular explanation for the similar phenotypes in CMT4B1 and CMT4B2. In *in vitro* and *in vivo* experiments, homodimeric MTMR2 interacts with homodimeric MTMR13 to form a tetrameric complex. This association dramatically increases the enzymatic activity of MTMR2 toward PtdIns3*P* and PtdIns(3,5)*P*_2_(72). Further regulation may be derived through redirecting the subcellular localization of the active enzyme MTMR2 by association with MTMR13 (72). In the absence of MTMR13, MTMR2 PI 3-phosphatase activity may be misregulated or mislocalized, leading to altered levels of PtdIns3*P* and/or PtdIns(3,5)*P*_2_ and subsequent membrane trafficking defects.

## PtdIns3*P* and PtdIns(3,5)*P*_2_ Dephosphorylation by MTM1 and X-Linked Centronuclear Myopathy

Numerous mutations of the myotubularin (*MTM1*) gene have been identified in patients affected with X-linked centronuclear myopathy, also called myotubular myopathy (XLCNM, OMIM 310400) (85). XLCNM is a very severe congenital myopathy associated with generalized muscle weakness and hypotonia at birth. The histopathology of skeletal muscle reveals small rounded fibers with central nuclei. This pattern is somewhat similar to the structure of fetal myotubes, while normal muscle fibers have peripherally located nuclei. About 200 different *MTM1* mutations have been reported in more than 300 unrelated families (86,87). They are distributed all along the gene and result in decreased or absent MTM1 in cell lines in the majority of cases (88). Almost all the truncating mutations are associated with a severe form of XLCNM, whereas missense mutations are sometimes associated with a milder form of the disease. The hypothesis that the muscle impairment reflects an altered PI regulation is supported by the characterization of a patient with a severe phenotype expressing an inactive MTM1 protein at normal level (59,88).

The *Mtm1* knockout mice recapitulate the histopathological signs of XLCNM and show a progressive myopathy starting a few weeks after birth, while muscle histology appears normal at birth (89). This suggests that the disorganized appearance of the muscle fibers is explained by a defect in structural maintenance rather than an impairment in myogenesis as previously hypothesized. As MTM1 homologs are mutated in CMT neuropathies, one could have thought that the muscle weakness reflected a nerve conduction defect. However, specific ablation of the gene in skeletal muscle showed that it is the primary tissue involved in the pathology (89). The *C. elegans* genome contains six *MTM* genes, three of which code for active enzymes. Null mutations in the closest homologue of *MTM1* are lethal, and *CeMtm-1* knockdown by RNA interference can correct the endocytic defect of *CeVps-34* mutant worms (22). This is consistent with a role of MTM1 in negative regulation of PtdIns3*P* levels. Moreover, mutations or knockdown of *mtm-3*, *mtm-6* and *mtm-9* (the latter encoding a catalytically inactive protein) lead to reduced fluid-phase uptake into coelomocytes (22,90). A recent study has shown a colocalization of MTM1 with RAB5 and RAB7 endosomes where it associates with the complex formed by PIK3C3 and PIK3R4 to presumably regulate the PtdIns3*P* endosomal pool (91). This observation has been made after saponin extraction of the cells, a method known to preserve membrane ultrastructure while extracting the MTM1 cytosolic pool. Stimulation of cells by the epidermal growth factor provokes a translocation of MTM1 to the late endosomal compartment dependent of PtdIns(3,5)*P*_2_ production (70). Artificial targeting of MTM1 to RAB5-containing endosomes induces microtubule-dependent tubularization of the endosomal network, suggesting that MTM1 at endosomes may impact on membrane remodeling and retrieval, probably through the function of its substrates, PtdIns3*P* and PtdIns(3,5)*P*_2_(92). A role of myotubularins at the plasma membrane is also suspected as overexpression of the MTM1 protein in mammalian cells leads to altered shape and plasma membrane projections and as MTM1 localizes to Ras-related C3 botulinum toxin substrate 1 (RAC1)-induced plasma membrane ruffles (93,94). Thus, MTM1 may play a role in membrane trafficking and remodeling although its specific sites of action are not yet defined. Defects in membrane remodeling could be the underlying cause of X-linked centronuclear myopathy.

## Tissue Specificity of Endosomal PIs Diseases

It is striking to note that ubiquitously expressed PI regulators acting in the same pathway are involved in pathologies affecting different tissues. This is especially noteworthy for MTM1 and MTMR2, which are two very close homologs (65% sequence identity) with similar enzymatic activity and which are implicated in diseases affecting two different tissues, skeletal muscles for MTM1 in XLCNM and Schwann cells in peripheral nerves for MTMR2 in CMT4B1, as emphasized by corresponding tissue-specific ablation experiments in both knockout mice. A first explanation could consist in differential regulation of expression between *MTM1* and *MTMR2*. Even if ubiquitous, *MTM1* is more expressed in skeletal muscles (94). MTM1 and MTMR2 present an inversed expression pattern during myogenesis: MTMR2 is expressed in the C2C12 mononucleated myoblast cells and decreases during the differentiation process, whereas MTM1 is increased at the RNA and protein level during myoblasts fusion in culture (88,93). Conversely, mouse MTMR2 was found to be particularly abundant in the neurons and Schwann cells of the peripheral nervous system (61,95). Suggestion for a specific protein function is based on the observation that subcellular localization differs between myotubularins. In transfected cells, MTMR2 is more concentrated around the nucleus compared with MTM1 (67,93). Such results suggest that different myotubularins may regulate different PI pools. A third possibility for tissue specificity could be interactions with tissue/cell-specific proteins. Thus, discs, large homolog 1 (DLG1) may be a Schwann cell-specific interactor that recruits and locally concentrates MTMR2 at paranodes to regulate membrane trafficking and membrane addition. Heterodimers of active and inactive myotubularins may also occur in specific tissues or cells (Schwann cells for MTMR2/MTMR13 and Sertoli cells for MTMR2/MTMR5), thus determining a tissue-specific activation or regulation of the active myotubularin partner. No muscle-specific partner is known for MTM1.

## Common Pathological Mechanism of Endosomal PI Diseases

CMT neuropathies types 4B1, 4B2 and 4J, X-linked centronuclear myopathy and a rare form of corneal dystrophy are caused by mutations in regulators of endosomal PIs. This pathway may also be involved in autophagy-related disorders and even in psychiatric diseases. PtdIns3*P* and PtdIns(3,5)*P*_2_ and their regulators all share a common function in membrane dynamics, with a possible implication in membrane fission and retrieval. Altered levels of PtdIns(3,5)*P*_2_ and PtdIns3*P*, following a loss of the MTMR2/MTMR13 complex in patients affected with CMT4B1/B2, may cause a defective transport of membranes between a storage compartment and myelin sheaths. Membrane fission is essential for membrane retrieval, and fission defects may also be the underlying cause of CMT dominant intermediate type B (CMTDIB) because of missense mutations in dynamin 2 (DNM2), a large GTPase involved in the tubulation of membranes and in the release of newly formed vesicles (96). Decreased levels of PtdIns(3,5)*P*_2_ caused by mutations in *PIP5K3* and *SAC3* genes in CFD and CMT4J patients may lead to a defect of membrane retrieval from late endosomal compartments, inducing vacuolization and perturbation of the late endosome–lysosome pathway. Abnormal regulation of the late endosome–lysosome pathway could also contribute to neurodegeneration in other forms of CMT neuropathies like the dominant axonal CMT2B associated with mutations in RAB7, a GTPase implicated in the transport of proteins to lysosomes and the dominant demyelinating neuropathy CMT1C caused by mutations in the E3 ubiquitin ligase lipopolysaccharide-induced TNF factor (LITAF) that targets membrane proteins for lysosomal degradation through a monoubiquitination mechanism (97,98). Likewise, XLCNM with mutations in *MTM1* may be caused by membrane remodeling alterations. This hypothesis is sustained by the findings that *DNM2* is also mutated in an autosomal dominant form of centronuclear myopathy and that mutations of amphiphysin 2 (*BIN1*) have recently been identified in patients with an autosomal recessive form of CNM (99,100). BIN1 is involved in membrane remodeling by sensing and promoting membrane curvature. MTM1, DNM2 and BIN1 may then be necessary for the remodeling and the delivery of membranes to T-tubules, which are plasma membrane invaginations implicated in excitation/contraction coupling in muscle fibers. Perturbation of T-tubules maintenance and/or endocytic membranes may represent one of the primary causes of CNM. However, the cause of the centralization of nuclei in the muscle fibers of CNM patients remains elusive. The fact that *MTM*-related proteins and *DNM2* are mutated in both centronuclear myopathies and CMT neuropathies further points to a common mechanism involving PIs and membrane retrieval in skeletal muscle and peripheral nerve maintenance.

## Conclusions

A significant amount of work has recently highlighted a role for the endosomal PIs in membrane retrieval and their implication in human diseases. This leads to the emerging concept that the associated diseases may be caused by altered membrane remodeling and retrieval at plasma membrane and membrane storage compartments. Additionally, PtdIns3*P* and PtdIns(3,5)*P*_2_ may also have roles independent of membrane traffic and may be found elsewhere than on endosomes. Questions remain about the mechanisms responsible for PtdIns3*P*- and PtdIns(3,5)*P*_2_-dependent membrane remodeling. Tissue-specific interactors of these endosomal PIs are probably involved in the tubulation and scission of membranes and in the transport of the resulting vesicles to correct locations in cells. Such interactors are interesting candidates for centronuclear myopathies and CMT neuropathies for which several responsible genes still remain to be identified.
